# The Role of Hydrolases in Biology and Xenobiotics Metabolism

**DOI:** 10.3390/ijms23094870

**Published:** 2022-04-28

**Authors:** Christophe Morisseau

**Affiliations:** Department of Entomology and Nematology, UCD Comprehensive Cancer Center, University of California, Davis, CA 95616, USA; chmorisseau@ucdavis.edu

Chemical exposure can profoundly affect our health, some being voluntary (food and drugs) and some involuntary (environmental contaminants). The metabolism of these chemicals is essential for their detoxification, elimination, and, sometimes, their activation. Of the numerous enzymes involved in the metabolism of xenobiotics, this Special Issue concentrates on hydrolases. Hydrolytic proteins are part of a heterogeneous group of enzymes that catalyze bond cleavages through reactions with water, the most abundant substrate in nature. Hydrolases can add water to numerous chemical functions (Figure 1) in an efficient way, involving them in the metabolism of numerous natural and man-made chemicals. Hydrolases have enzyme commission numbers starting with three.

Contrary to CYP450, which are more “slow and steady wins the race” enzymes [1], hydrolases have a fast substrate turnover. Thus, their action is generally limited by substrate selectivity, substrate availability, and/or inhibition. Because of their fast catalytic activity, the significant reduction in metabolism by hydrolases involves covalent or tight binding inhibitors. The usage of such inhibitors in animal models, as well as transgenic animals [2], has led to recent novel findings demonstrating biological roles for hydrolases, implicating them in the regulation of cardiovascular diseases, inflammatory responses, neurologic diseases, energy metabolism, etc. [3,4].

In light of these new findings, this Special Issue focuses on both the basic science and translational research of the biological roles of hydrolases in mammals, as well as their role in the metabolism of toxins and natural products. The Special Issue contains five reviews and nine research articles concerning the role of four substrate classes of hydrolases, as well as artificial amyloid hydrolases.

## 1. Epoxide Hydrolase

In this special issue, a review by *Gautheron et Jéru* [5] summarizes the contribution of epoxide hydrolase (EH) in mammals to the metabolism of endogenous bioactive lipid epoxides related to several diseases. In mammals, soluble (sEH) and microsomal (mEH) epoxide hydrolases appear to have complementary roles in metabolizing lipid epoxides in terms of substrate regioselectivity, but also the presence of the two enzymes in different tissues, highlighting balancing roles in disease states (*Morisseau* et al. [6]). This is illustrated in several research papers investigating the role of sEH and epoxy fatty acids in several animal models of diseases. The inhibition of sEH by the use of a natural product has antihypertensive effects, yielding renal and cardiac protection (*Elbarbry* et al. [7]). This later effect could be beneficial for reducing the risk of myocardial infarction in middle-aged women, who have higher levels of sEH (*Jamieson* et al. [8]). Three research papers (*Overby* et al.; *Yang* et al.; *Davis* et al. [9,10,11]) highlight the deleterious effect of the sEH hydrolysis of epoxy-fatty acids in diet-induced diabetes and obesity. A high-fat diet increases sEH activity in various tissues around the body; in the brain, these increases abolish the protective effect of sEH inhibition following a stroke, while in brown adipose, sEH inhibition increases lipid metabolism and reduces circulating triglycerides. This latter effect seems to result from higher levels of 17,18-EEQ from EPA and 19,20-EDP from DHA that increase the BAT beneficial metabolic function. On the other hand, the lack of sEH activity seems to promote the growth of breast cancer, although not its metastasis (*Kesavan* et al. [12]).

## 2. Glycoside Hydrolase

Glycoside hydrolases catalyze the hydrolysis of glycosidic bonds (ether bonds) in complex sugars. These enzymes are ubiquitous and well known for their role in the biomass metabolism. Here, *Rafiei* et al. [13] reviewed the current knowledge regarding fungal plant pathogens that utilize glycoside hydrolases to perforate the cell wall of plant cells to successfully invade the host. Some of these proteins trigger plant immune responses.

## 3. Phosphatase

Phosphatases are well known to oppose the action of kinases by removing phosphate groups from numerous substrates. The regulation of the phosphorylation of protein is used at the cellular level to regulate biological action and/or catabolism. Here, a research article by *Chrabąszczewska* et al. [14] delves deep into the substrate preference of the human Nudt16 protein (hNudt16). This hydrolase metabolizes nucleoside diphosphate, and is involved in the N cellular metabolism, homeostasis, and mRNA processing. Separately, a review by *Roychoudhury and Hegde* [15] brings us up to date with the eye absent protein (EYA), a haloacid dehalogenase (HAD) tyrosine phosphatase, with a mechanism different from other enzymes in the same family. EYAs are at least involved in DNA repair, play a critical role in normal cell development, and contribute to disease pathologies.

## 4. Proteosome

Proteasomes are cellular complexes of proteolytic enzymes (proteases) that regulate protein levels, especially misfolded proteins. Besides this constitutive role, proteasomes have roles in cell cycle regulation and cellular survival from stress. Recently, proteasomes have been the target of anticancer approaches illustrated by the approval of selective proteosome inhibitors for the treatment of multiple myeloma. Here, *Wang* et al. [16] review the current knowledge regarding proteosome inhibitors, especially their metabolism and ADME properties.

## 5. Amyloid Hydrolase

Amyloids are protein aggregates that have been associated with the onset of several diseases, such as Alzheimer’s or Parkinson’s disease. Nevertheless, amyloid aggregates represent a new kind of protein fold that has recently been utilized for the design of novel enzymatic activities. In their review, *Duran-Meza and Diaz-Espinoza* [17] summarize the current literature on devising esterases, phospho-esterases, and di-phosphohydrolases from amyloid aggregates, offering a novel alternative to natural hydrolase.

## 6. Conclusions

The articles and reviews in this Special Issue highlight how the vision of hydrolases has shifted from a role in metabolism to a more complex role in cellular signaling and regulation. This is underscored by the fact that hydrolases are now the target of drugs for several disease treatments, promising more research on this great family of enzymes.

## Figures and Tables

**Figure 1:**
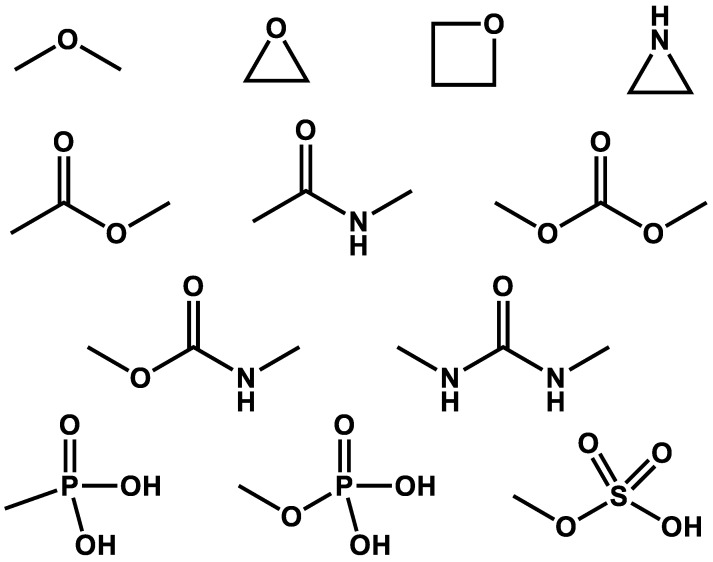
Chemical functions targeted by hydrolases.
